# Resistive Switching of Sub-10 nm TiO_2_ Nanoparticle Self-Assembled Monolayers

**DOI:** 10.3390/nano7110370

**Published:** 2017-11-04

**Authors:** Dirk Oliver Schmidt, Nicolas Raab, Michael Noyong, Venugopal Santhanam, Regina Dittmann, Ulrich Simon

**Affiliations:** 1 JARA-FIT, 52056 Aachen, Germany; oliver.schmidt@ac.rwth-aachen.de (D.O.S.); michael.noyong@ac.rwth-aachen.de (M.N.); 2 Institute of Inorganic Chemistry, RWTH Aachen University, 52074 Aachen, Germany; 3 JARA-FIT, 52425 Jülich, Germany; n.raab@gmx.net (N.R.); r.dittmann@fz-juelich.de (R.D.); 4 Peter Grünberg Institut 7, Forschungszentrum Jülich GmbH, 52428 Jülich, Germany; 5 Department of Chemical Engineering, Indian Institute of Science, Bangalore 560012, India; venu@chemeng.iisc.ernet.in

**Keywords:** TiO_2_ nanoparticles, self-assembly, resistive switching

## Abstract

Resistively switching devices are promising candidates for the next generation of non-volatile data memories. Such devices are up to now fabricated mainly by means of top-down approaches that apply thin films sandwiched between electrodes. Recent works have demonstrated that resistive switching (RS) is also feasible on chemically synthesized nanoparticles (NPs) in the 50 nm range. Following this concept, we developed this approach further to the sub-10 nm range. In this work, we report RS of sub-10 nm TiO_2_ NPs that were self-assembled into monolayers and transferred onto metallic substrates. We electrically characterized these monolayers in regard to their RS properties by means of a nanorobotics system in a scanning electron microscope, and found features typical of bipolar resistive switching.

## 1. Introduction

The astounding developments in information technology over the last few decades reliably obeyed Moore’s law [1,2]. However, this predicted trend of miniaturization is coming to an end due to physical limitations [3]. At the same time, an increasing demand for digital data storage is anticipated, which will require new, non-volatile data storage technologies in the near future.

Resistive random access memories (RRAM) are promising candidates for data storage applications [4,5]. They rely on resistive switching (RS), which results from a resistance change of a functional layer sandwiched between metal electrodes. RRAM devices are typically composed of a metal–insulator–metal layer structure, mainly in the form of thin films that are structured by means of lithographic (top–down) techniques. As an alternative approach, nanoparticle (NP) thin films formed via chemical synthesis and assembly can be utilized as a functional layer in RS devices. Such a bottom–up approach in principle allows the fabrication of cell dimensions that exceed the size limits of top–down approaches [6]. From a technological point of view, NPs can be synthesized via inexpensive methods and under mild reaction conditions [7]. Subsequently, the NPs can be deposited on the electrodes using solution-based techniques that are suitable for organic or polymeric substrates, thus leading to flexible memory devices [8].

The resistive switching of NPs is often investigated in a configuration that is similar to conventional thin film cells, wherein NP assemblies are the functional layer of the device. Typically, most of these RS cells based on NP assemblies were fabricated as follows: firstly, NPs are deposited on a bottom electrode via spin-coating or dip-coating methods, which enable control of the NP assembly thickness. Secondly, top electrodes are deposited on the NP assemblies. One of the first reports on RS of a NP assembly as a functional layer utilizing Fe_3_O_4_ NPs was given by Kim et al. in 2009 [9]. In the following years, the RS of iron oxide-based NPs were further investigated, e.g., consisting of Fe_2_O_3_ NP assemblies [8,10,11], Pt-Fe_2_O_3_ core–shell NP assemblies [12], or of mixed Pt–Fe_2_O_3_ core–shell/Fe_2_O_3_ NP assemblies [13]. Besides iron oxide NPs, RS behavior was also reported for CdS NPs [7], CeO_2_ nanocubes [14,15], BaTiO_3_ NPs [16], ZnO NPs [17], NiO NPs [18], Ge–GeO_x_ nanowires (NWs) [19], and for In_2_O_3_ nanorods [20]. The RS of assemblies consisting of spherical 3 nm TiO_x_ NPs was demonstrated by Goren et al. in a Co–TiO_x_ NPs–Co structure [21]. The TiO_x_ NPs were synthesized by a sol–gel method, and the as-synthesized NPs were amorphous. NP films with a thickness of 55 nm were prepared by spin-coating, and the cells showed bipolar resistive switching (BRS). The NP devices were compared with a TiO_x_ thin film device, and while the film device exhibited only switching at one interface, the authors report switching at both interfaces for the NP device.

However, despite the very small size of the individual NPs (e.g., 3 nm), the thickness of the NP assemblies is often quite high, and thicker than typical thin film structures. As an exception, Uenuma et al. demonstrated BRS for a monolayer of 6 nm magnetite NPs in a metal–NPs–metal structure [22].

Recently, we reported the RS of individual TiO_2_ NPs with sizes of approximately 350 nm as well as 50 nm [23]. In order to continue down-scaling the RS devices composed of TiO_2_ NPs, we chemically synthesized sub-10 nm TiO_2_ NPs by a solvothermal method. We chose TiO_2_ as a model material because its RS properties are investigated in single crystals [24] as well as thin films [25,26]. Furthermore, the complementary metal-oxide-semiconductor (CMOS) compatibility of TiO_2_ [27], as well as the abundance of Ti in the earth’s crust [28], ensures its economic viability. The immobilization of the TiO_2_ NPs acting as switching units is necessary in order to integrate NPs into resistive switching devices. Therefore, the self-assembly of NPs into a well-ordered, hexagonally packed monolayer is desirable. In the literature, different methods are reported to obtain self-assembled NP monolayer. One is the drop-drying of a colloidal solution [29], which is assisted by an electric field [30] or by molecule interactions [31]. Another is by spreading a colloidal solution of hydrophobic NPs onto a water surface [32,33,34]. A review summarizing the various methods can be found in reference [35]. However, to the best of our knowledge, up until now, the self-assembly of sub-10 nm TiO_2_ NPs into monolayer films has not been reported.

In this paper, we present the synthesis of sub-10 nm TiO_2_ NPs and their characterization by means of powder X-ray diffraction (XRD) as well as transmission electron microscopy (TEM). Self-assembly experiments were performed in order to obtain hexagonally close-packed TiO_2_ NP films on a water surface, and we obtained TiO_2_ NP monolayer films with lateral dimensions of 1 µm^2^. We transferred the self-assembled films to planar Pt–Ir surfaces, which function as bottom electrodes, via two different approaches. The transferred films were characterized using a scanning electron microscope (SEM), atomic force microscope (AFM), and transmission electron microscope (TEM). We performed localized electrical measurements by means of a nanorobotics setup in a SEM, as well as by means of local conductive atomic force microscopy (LC-AFM). Finally, we investigated the RS properties of the films in a SEM.

## 2. Results

### 2.1. Solvothermal Synthesis of TiO_2_ Nanoparticles

In order to synthesize TiO_2_ NPs with a diameter of sub-10 nm, we adapted the solvothermal synthesis methods of Dinh et al. [36]. Titanium butoxide was used as titanium precursor, oleylamine and oleic acid were used as capping agents. The ratio of the two capping ligands allowed the authors to control the particle morphology. Rhombic NP shapes were obtained with a titanium butoxide/oleic acid/oleylamine ratio of 1:4:6; truncated rhombic NP shapes were obtained with a ratio of 1:5:5, and spherical NP shapes were obtained with a ratio of 1:6:4. With regard to the envisaged self-assembly of the TiO_2_ NPs as monolayers, as densely as possible, the spherical morphology is desirable, as it allows for a hexagonal close packing, thereby covering approximately 91% of the available surface [37].

**Figure 1:**
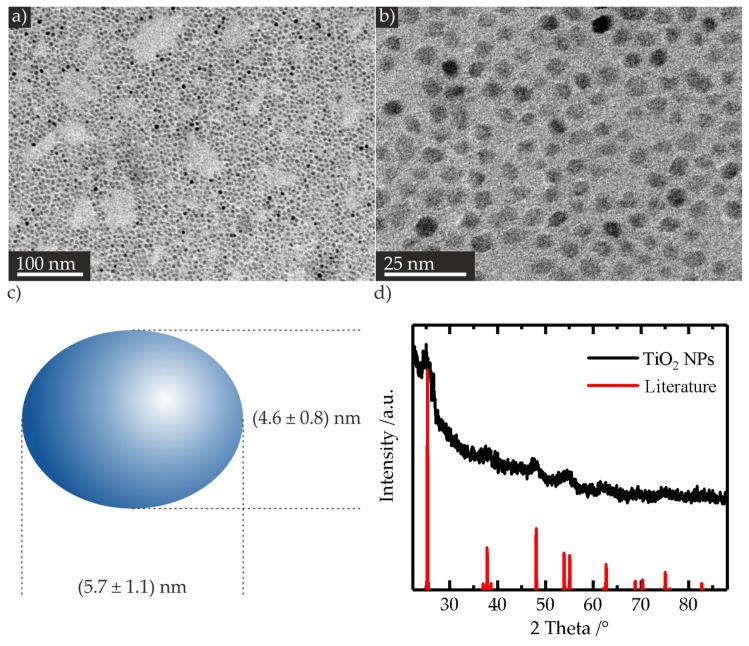
Representative TEM images of the synthesized TiO_2_ nanoparticles (NPs) (**a**,**b**). Schematic illustration of the NP with the corresponding mean longitudinal and transversal (**c**). Powder XRD reflection patterns of the NPs (black), and simulated literature anatase reflection patterns (red) (**d**).

In a series of syntheses, we obtained the highest amount of spherical shaped NPs with a titanium butoxide/oleic acid/oleylamine molar ratio of 1:3:2, and avoiding ethanol solvent. The synthesized TiO_2_ NPs are shown in Figure 1a,b. The ratio of spherical to non-spherical shaped NPs was ca. 27:1, meaning that 96% of the yielded NPs presented a spherical morphology. A mean particle longitudinal of (5.7 ± 1.1) nm and a mean particle transversal of (4.6 ± 0.8) nm were determined (Figure 1c; for the corresponding histograms, see Figure S1), which implied a nearly spherical morphology. Recorded powder XRD reflection patterns of the obtained NPs showed broadened reflexes, according to the minute particles’ size, and matched the simulated anatase reflection patterns (Figure 1d) [38].

### 2.2. Formation of Self-Assembled TiO_2_ Nanoparticle Monolayers

**Figure 2:**
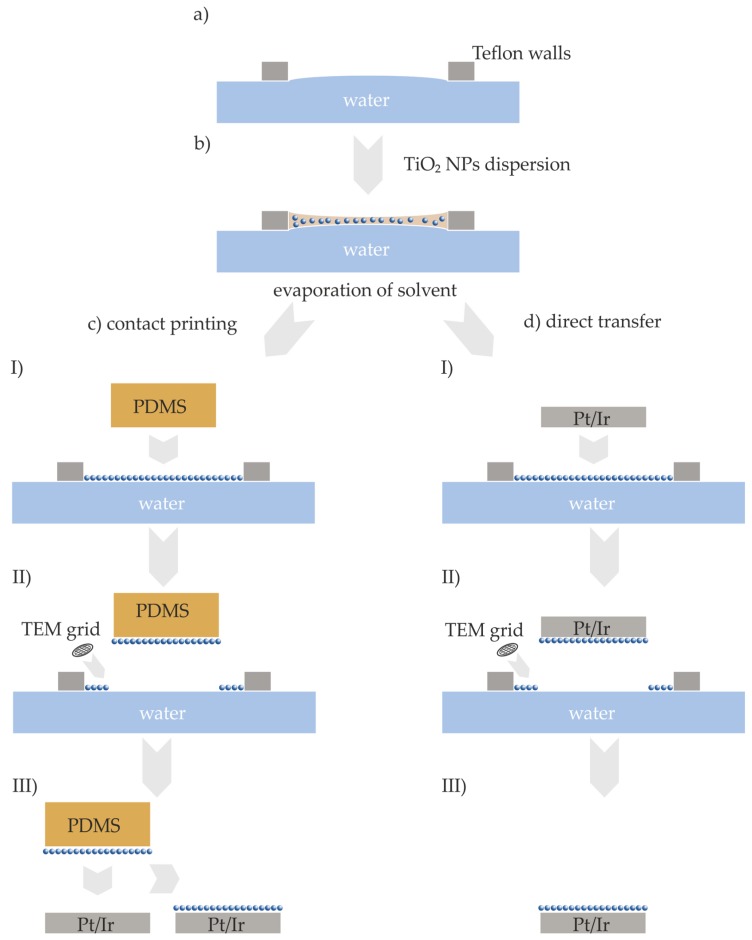
Schematic drawing for the self-assembly of a TiO_2_ NP monolayer (not drawn to scale) (**a**,**b**), for the two-step, microcontact printing method (**c** I to III), and for the one-step method for the direct transfer of self-assembled TiO_2_ films (**d** I to III).

In order to obtain TiO_2_ NP monolayer films, we followed the method of Santhanam et al., in which the authors applied for the monolayer formation of hydrophobic gold NPs [33]. Briefly, the synthesized sub-10 nm TiO_2_ NPs were dispersed in an organic solvent and were dropped on a water surface with a controlled surface curvature. Due to the evaporation of the organic solvent, a self-assembled monolayer was formed on the water surface. In order to perform TEM investigations of the self-assembled film immobilized at the water/air surface, the surface was touched with a carbon coated TEM grid. A schematic drawing of the method is shown in Figure 2a,b.

The formation of well-ordered monolayer films is challenging, because it depends on the following experimental parameters. First, the NPs have to be spherical and monodisperse; otherwise, a hexagonally close packing of the NPs is not possible. Second, the TiO_2_ NPs need to be functionalized with hydrophobic ligands for the formation of a stable colloidal dispersion in organic solvents. Furthermore, the concentration of TiO_2_ NPs in the organic solvent influences the self-assembly. In this context, too low concentrations lead to small and isolated regions of closed packed NPs, whereas too high concentrations lead to multilayers of NPs. Furthermore, the organic solvent itself has to fulfill certain requirements. Most importantly, the solvent must allow for a stable dispersion of TiO_2_ NPs, and the density of the solvent must be lower than that of water. Finally, the evaporation rate of the chosen solvent determined by its volatility is crucial, since too fast as well as too slow evaporation rates induce the formation of multilayers instead of monolayers. Mixtures of different solvents can be used to precisely adjust the properties and meet these requirements. Additionally, the rate of evaporation of the organic solvents depends upon the air velocity and the temperature in the laboratory hood, which thus also influence the NP monolayer formation. In the scope of this work, the colloid concentration, the solvent composition, and the evaporation rate were investigated. We performed the experiments under air/ambient conditions.

**Figure 3:**
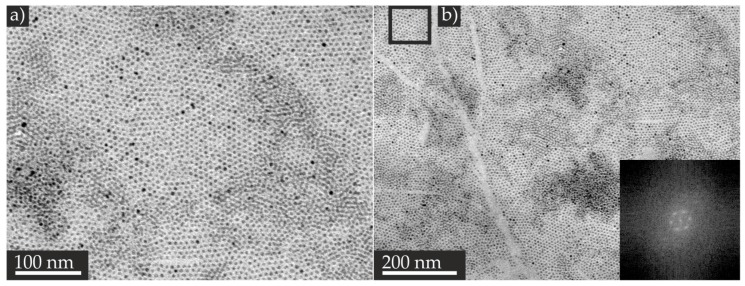
Representative TEM images of the self-assembled TiO_2_ NP film with decreasing magnification (**a**,**b**). The inset in (**b**) shows the fast Fourier transformation of the black highlighted area of the monolayer.

In a series of experiments, we obtained the largest continuous self-assembled TiO_2_ NP monolayer film with NPs presenting a mean particle longitudinal of (5.7 ± 1.1) nm and a mean particle transversal of (4.6 ± 0.8) nm, as well as a spherical NP to irregular-shaped NP ratio of 27:1 (see Figure 1). Since the as-synthesized TiO_2_ ligands were functionalized with oleic acid and oleylamine as hydrophobic ligands, no additional ligand exchange reactions were necessary. We prepared the self-assembled film with a solvent mixture of pentane/dichloromethane 3:1, 0.56 g/mL TiO_2_ NPs concentration, and the addition of 0.0076 mol/L oleylamine solution in hexane to the dispersion. The corresponding TEM images (Figure 3a,b) revealed that an area of approximately 1 µm^2^ was covered with mostly a monolayer of TiO_2_ NPs. The dense, close packing is clearly visible in the images. Fast Fourier transformation was performed in these regions with help of the Software ImageJ (Version 1.43u) showing the reconstructed hexagonal patterns (see Figure 3b, inset). The center-to-center spacing of the NPs amounted to ca. (9 ± 1) nm, which corresponds to the dimensions of the NPs, plus the approximately 2 nm length of the oleic acid and oleylamine ligands, assuming that a monolayer of the ligands has formed on the TiO_2_ NP surface. A similar spacing of 2 nm was reported by Sun et al. for monodispersed, hexagonally packed FePt NPs capped with oleic acid and oleylamine [29]. Additionally, several patches of bi- and multilayers were formed, as indicated by the areas showing a lower brightness in the TEM images compared with those of the TiO_2_ NP monolayers (Figure 3a,b). The formation of well-ordered monolayers only took place in confined regions, preferably at the edges of multilayers. The formation of the multilayer domains can be mainly attributed to fluctuations along the retracting contact line during the evaporation of the solvent, as well as due to tearing of the film during the transfer process. Experiments revealed that the adjustable parameters of colloid concentration and solvent could be controlled well. However, the evaporation rate could not be fully managed due to the random temperature and air velocity of the fume hood. Nevertheless, in this work, we produced a well-ordered, self-assembled TiO_2_ NP monolayer, and the observed dimensions of the TiO_2_ NP monolayers are sufficient enough for RS experiments performed by means of the nanorobotics setup in SEM.

### 2.3. Preparation of Resistive Switching Devices

The self-assembled TiO_2_ NP films were formed on a water surface, and had to be transferred onto a metallic surface as the bottom electrode in order to allow the investigation of their RS behavior. A 1 cm^2^ silicon wafer with a native oxide layer, which was coated with a homogenous 180 nm thick Pt/Ir alloy (80% Pt, 20% Ir) metal film, was used as the support. In order to transfer the assembled TiO_2_ NP films to a metal surface, the film was carefully brought into contact with a polydimethylsiloxane (PDMS) stamp, or directly with a Pt/Ir surface. A schematic drawing of the two methods is illustrated in Figure 2.

**Figure 4:**
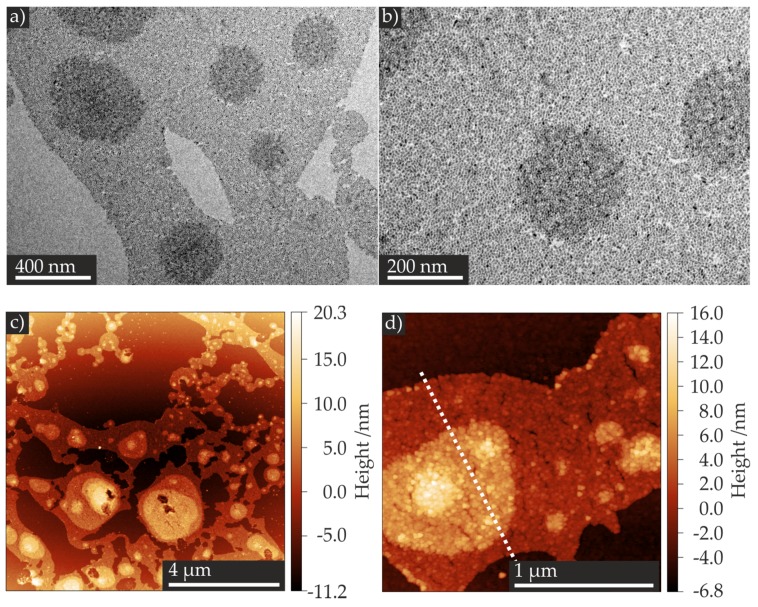

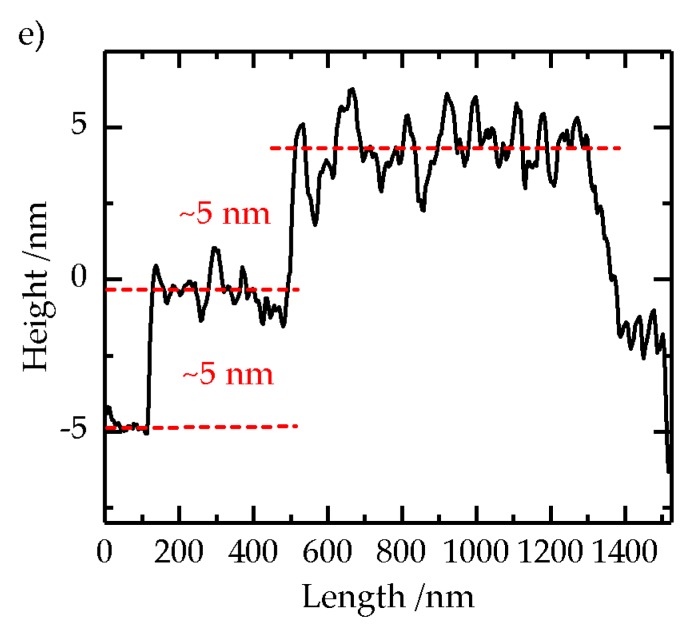
Exemplary TEM images of the self-assembled TiO_2_ NP film (**a**,**b**). Tapping mode atomic force microscope (AFM) images of the TiO_2_ NP film transferred by the microcontact printing method onto a Pt/Ir surface (**c**,**d**). Corresponding height profile (**e**) taken along the white line in (**d**) showing height differences of ca. 5 nm.

The two-step method was performed following a published microcontact printing procedure [39]. In contrast to the direct, one-step method, the TiO_2_ NP film was first transferred to a stamp, and afterwards the TiO_2_ NP film was printed to any desired surface. The application of stamps with nanoscaled features would allow for the preparation of nanoscaled TiO_2_ NP patterned surfaces as resistive switching devices [39]. The TiO_2_ NP film shown in the TEM images (Figure 4) was prepared with NPs that had a mean longitudinal of (7.9 ± 2.2) nm, a mean transversal of (4.8 ± 0.8) nm, and a spherical NP to non-spherical shaped NP ratio of 5:1 (for the characterization of these NPs, see Figure S2). We lifted the TiO_2_ NP film from the water surface with a planar polydimethylsiloxane (PDMS) stamp (Figure 2c I and II). After the evaporation of the residual water droplet, the dried films on the planar side of the PDMS stamp were transferred to a Pt/Ir surface by pressing the stamp onto the surface (Figure 2c III). We characterized the formed film via TEM (Figure 4a,b), and the transferred film via AFM (Figure 4c,d). The TEM images as well as AFM images show similar TiO_2_ NP mono-, bi-, and multilayers, as well as voids between the layers. Since we observed no macroscopic wrinkles or cracks in the TEM images, we conclude that the self-assembled TiO_2_ NP film was transferred from the water surface to the carbon film of the TEM substrate without any changes of the film, at least in the dimensions shown in the TEM images. The recorded height profile revealed a variation of height of approximately 5 nm (Figure 4e), which corresponds well to the dimensions of the NPs determined by TEM analysis. Based on the resemblance of AFM and TEM images, we concluded that the self-assembled TiO_2_ NP film was successfully transferred onto the Pt/Ir surface. We obtained similar results for the one-step method (see Figure 2d I to III). For detailed results, see Figure S3 in the Supporting Information. Hence, we successfully prepared RS devices composed of TiO_2_ NP films by means of two different methods for the subsequent electrical characterization.

### 2.4. Electrical Characterization

**Figure 5:**
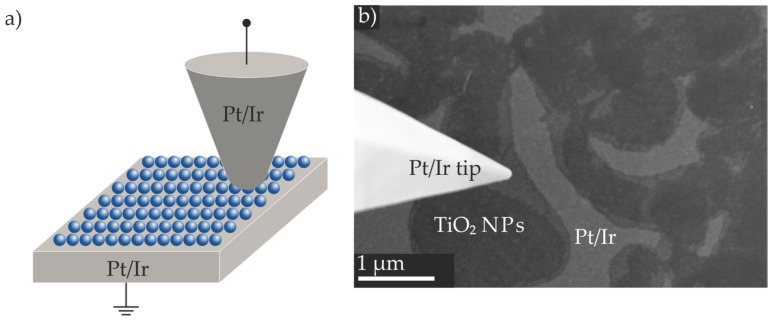
Schematic illustrations of the (Pt/Ir)/TiO_2_ NP film/(Pt/Ir tip) device (**a**). Exemplary SEM image of a TiO_2_ NP film on the Pt/Ir surface transferred by the one-step method; on the left hand side, the Pt/Ir coated tip electrode is visible (**b**) (contrast of the SEM image was increased after the measurement; for the original SEM image, see Figure S4).

We performed RS experiments by means of a nanorobotics system for local in situ electrical measurements in SEM [40]. Prior to the in situ electrical characterization in SEM, we performed an oxygen plasma cleaning step with the (Pt/Ir)/TiO_2_ NP film substrates to widely remove the oleylamine, as well as the oleic acid ligands. As top electrodes, we utilized Pt/Ir coated AFM probes with a radius of curvature of approximately 100 nm, and a special elongated tip in the front part of the cantilever; thus, they are visible from the top in the SEM. This setup enables the flexible addressing of certain locations on the thin films. While the voltage was applied to the tip, the planar Pt/Ir bottom electrode was set to ground. We monitored the movement of the tip on the NP films, as well as the structural changes of the tip during the resistive switching experiments. After one measurement, the tip electrode was lifted off and moved to the next point of interest, which allowed successive characterization under identical experimental conditions [40]. Schematic illustrations of the experimental setup and an exemplary SEM image are displayed in Figure 5a,b, respectively.

Directly before the experiment, we cleaned the SEM chamber, the measurement tips, and the TiO_2_ NP films with Ar plasma to further eliminate contaminations. Due to NP diameters below 10 nm and weak material contrast, individual TiO_2_ NPs immobilized on the Pt/Ir surface could not be resolved in SEM during the electrical characterization, which requires a large working distance due to the presence of the tip. However, comparing the AFM, TEM, and SEM images, we assume that the bright regions are the Pt/Ir surface (Figure 5b). Furthermore, we assume that the areas exhibiting a slightly lower brightness correspond to TiO_2_ NP monolayers, while the darkest areas correspond to TiO_2_ NP multilayers. At the left hand side of the SEM image, the measurement probe that was brought into mechanical contact with a TiO_2_ NP monolayer is visible.

In order to test different areas, brighter and darker regions discernible in the SEM images (see Figure 5b) were contacted with the Pt/Ir tip. The recorded *I–V* curves showed a linear behavior and resistances of ca. 300 Ω, which matched the resistances determined by addressing a pristine metallic surface. Hence, the electrical characterization confirmed that the bright regions do correspond to the Pt/Ir bottom electrode. By positioning the tip on regions exhibiting a lower brightness, strictly non-linear *I–V* curves revealing a high resistance were recorded. These regions are identified as corresponding as expected to the TiO_2_ NP layer. Therefore, during the in situ electrical characterization in SEM, SEM images and electrical responses allowed for facile differentiation between the Pt/Ir bottom electrode and the TiO_2_ NP layer.

In the SEM images (Figure 5b), stepwise brightness differences are visible within the TiO_2_ NP layer. Based on the TEM and AFM analysis results, the TiO_2_ NP layer with a higher brightness is assumed to correspond to a monolayer, while the TiO_2_ NP layer with a lower brightness is assumed to correspond to a multilayer. Different spots of the TiO_2_ NP monolayer or multilayer were brought into contact with the tip, and non-linear *I–V* curves without any hysteretic behavior were recorded. The tip diameter of the Pt/Ir coated measurement probes was approximately 100 nm. Hence, multiple sub-10 nm TiO_2_ NPs were simultaneously addressed. However, we did not find a clear dependence between layer thickness and resistance. In multiple layers, the absolute number of particles that contributed to the conducting path varied. Moreover, different numbers of resistances by each particle in series and in parallel lead to varying overall resistance.

**Figure 6:**
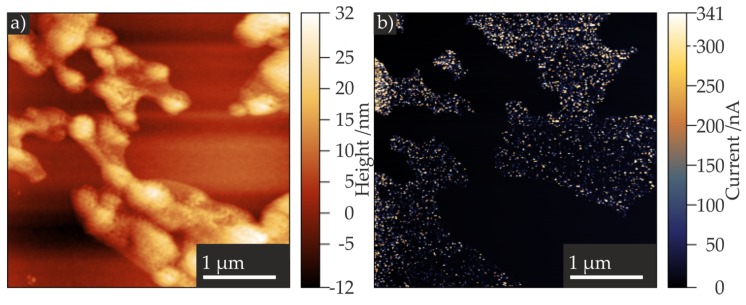
Contact mode AFM images of a TiO_2_ NP film on a Pt/Ir surface (**a**) and corresponding distribution of the current (**b**), scanned simultaneously with the topography.

Additionally, we performed LC-AFM measurements. The LC-AFM allows the simultaneous measurement of topography and current through the sample, and is operated in the contact mode to record the current distribution of the scanned region. We utilized conductive diamond tips AppNano Doped Diamond with a radius of curvature of 100–300 nm for the experiments. In order to perform the electrical measurements without inducing a resistance change of the NPs, a voltage of 20 mV was applied to the tip, and the topography and the current were recorded simultaneously. The contact mode topography image (Figure 6a) revealed TiO_2_ mono-, bi-, and multilayers similar to those observed in tapping mode. In the current mapping image (Figure 6b), the TiO_2_ NP layer can be clearly distinguished from the Pt/Ir surface, as the latter exhibited currents ranging from approximately 100 nA to 340 nA, while the areas with TiO_2_ NP layers exhibited no current flow.

These findings by means of SEM and LC-AFM are in agreement with the metallic, highly conducting character of the Pt/Ir surface and the insulating character of the anatase TiO_2_ NPs [41].

**Figure 7:**
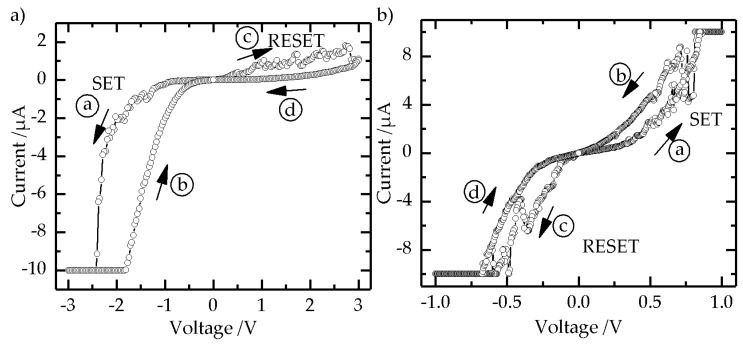
Two *I–V* curves recorded on different TiO_2_ NP monolayers exhibiting bipolar resistive switching (BRS) behavior (**a**,**b**) (arrows and small letters depict voltage sweep sequence).

In order to study the RS behavior of our devices in the SEM, we applied write voltage sweeps from 0 V → *X* V → 0 V → *X* V → 0 V, or from 0 V → −*X* V → 0 V → *X* V → 0 V. We set a current compliance of 1 µA up to 10 µA to protect the TiO_2_ NP layer, as well as the metal coating of the measurement tips. We identified a current compliance of 10 µA to be suitable for the resistive switching experiments. The *I–V* curve shown in Figure 7a was recorded on a TiO_2_ NP monolayer area contacted by the measurement tip. The *I–V* curve showed a typical BRS behavior, with a SET process of the device from the high resistance state (HRS) into the low resistance state (LRS) at a voltage of ca. −2.5 V. The RESET process, the switching of the device from the LRS to the HRS, took place over a voltage range from 1.0 V to approximately 2.8 V, and switched the device back into the HRS. Hence, for the *I*–*V* curves shown in Figure 7a, we observed the counter eightwise switching polarity. The hysteresis and the current are larger at a negative voltage polarity compared with the positive voltage polarity. The recorded *I–V* curve shown in Figure 7b, which was also recorded on a TiO_2_ NP monolayer, demonstrated BRS behavior exhibiting the SET process at a positive voltage, and the RESET process at a negative polarity; hence, eightwise switching polarity is observed. In general, the switching polarity of a BRS device is determined by a microstructurally asymmetric cell design, or a voltage/current-controlled electroforming process. The underlying switching mechanism for valence change memories is generally explained by a formation and rupture of a conductive filament inside the insulating TiO_2_ matrix due to the redistribution of oxygen vacancies under an applied electric field, and the effect of Joule heating [42]. This gives rise to a resistance hysteresis exhibiting the counter eightwise polarity [43]. The resistance hysteresis showing an “eightwise” switching polarity was recently investigated in SrTiO_3_ thins films by means of detailed in situ TEM analysis. Electrochemical oxygen evolution and oxygen reduction reactions were found to be responsible for the resistance change [44]. In order to decipher the underlying switching mechanism for our TiO_2_ NP devices, comparable elaborate analysis would be required, which goes far beyond the scope of this paper. For LC-AFM measurements, the switching polarity of a Fe:SrTiO_3_ film could be adjusted via the switching voltage [43]. The measurement tip was in contact with the TiO_2_ NP layer as briefly as possible to keep the thermal drift and creep effects in the piezoelectric control elements of the nanorobotics setup, as well as the specimen stage, as low as possible during the electrical characterization. Nonetheless, the position and the contact force of the tip changed during the application of voltage sweeps. Hence, the switching polarity could not be controlled in our experiments.

The device exhibiting the *I–V* curve, as shown in Figure 7b, was switched between 1 V and −1 V. The current reached the current compliance of 10 µA at both voltage polarities. Upon repeating the voltage sweep, the size of the hysteresis and the current values changed. After three cycles, the BRS (see Figure S5a) behavior was no longer observed. Instead, the current showed linear dependence on the applied voltage (see Figure S5b). 

Additionally, in some cases, we observed a structural change of the contacted TiO_2_ NP layer via SEM (see Figure S6). It is possible that the high voltage and current, accompanied by Joule heating, induced the structural change, leading to a direct contact between the measurement tip and the Pt/Ir surface, and thereby resulting in the observed linear behavior. During the prolonged application of voltage sweep, the position and the contact force of the tip changed due to the thermal drift and creep effects in the piezoelectric control elements of the nanorobotics setup and specimen stage, which also resulted in a possible direct contact. However, for most of the measurements, we observed a short circuit, although in the SEM images no clear damage of the NP layer was visible. Alternatively, it may be possible that individual sub-10 nm TiO_2_ NPs could be reduced to a better conducting state, e.g., Ti_4_O_7_ Magnéli phases [45]. These phases show metallic conductivity, and thus may be responsible for the observed linear behavior of the *I–V* curves [42]. However, a detailed analysis to identify Magnéli phases would go far beyond the scope of this work. We found during the measurements that voltages above ±3 V tend to cause a short circuit of the RS devices. In total, we electrically characterized 14 spots of a TiO_2_ NP monolayer, as well as 12 spots of a TiO_2_ NP multilayer. Overall, six spots showed a BRS behavior.

### 2.5. Summary

In order to obtain sub-10 nm resistive switching units, we synthesized TiO_2_ NPs with a size below 10 nm by a solvothermal method. Self-assembly of a TiO_2_ NP monolayer film on a water surface was prepared following the method of Santhanam et al. We achieved dense packed monolayers in an area of 1 µm^2^, which up until now had not been accomplished elsewhere. Since the TiO_2_ NP films were prepared on a water surface, they had to be transferred to metallic surfaces in order to subsequently electrically characterize the NPs. We successfully executed a one-step method, as well as a two-step microcontact printing method to transfer the self-assembled film to Pt/Ir surfaces that acted as bottom electrodes during resistive switching experiments. The microcontact printing method especially paves the way for the preparation of structured devices. The electrical characterization of the self-assembled TiO_2_ NP films on Pt/Ir bottom electrodes was performed by means of the nanorobotics setup SEM, as well as by LC-AFM. With both methods, we could unambiguously distinguish the Pt/Ir surface from the TiO_2_ NP layer by their different electrical response. BRS-like behavior of the TiO_2_ NP monolayer films was observed in SEM.

## 3. Materials and Methods 

**Solvothermal synthesis.** TiO_2_ NPs were synthesized modifying a solvothermal approach known from the literature, which allows the control of the NP morphology by adjusting the molar ratio of the Ti(OBu)_4_/oleic acid/oleylamine (TB/OA/OAM) [36]. Oleic acid was purchased from Sigma Aldrich (Taufkirchen, Germany), oleylamine (C-18 content 80%–90%) from Acros Organics (Schwerte, Germany, and titanium butoxide (Ti(OBu)_4_) (97% purity) from Sigma Aldrich (Taufkirchen, Germany). Typically, OAM and OA, as well as ethanol (EtOH) (absolute, 99% purity, Fisher Chemicals, Schwerte, Germany), were added in the Teflon inset, and stirred with a magnetic stirrer. After the addition of TB, stirring was continued for 10 min. The vessel was sealed with a Teflon lid, and set into a stainless steel autoclave. The autoclave was heated in a furnace to the reaction temperature for a determined time. Afterwards, the autoclave was cooled down to room temperature, and the product was transferred into 50 mL polystyrene tubes. Particle solutions were centrifuged and purified by washing with EtOH by suspension and centrifugation cycles. The obtained powder was dried at room temperature and characterized by powder XRD and TEM measurements. For the transmission electron microscopy analysis, the samples were dispersed in *n*-hexane (Riedel de Haen, 99% purity, Seelze, Germany) in an ultrasonic bath. The suspension was deposited on a carbon film copper mesh and dried. Best results were obtained with 1.44 mL TB (4.25 mmol), 4.04 mL OA (12.73 mmol), 2.79 mL OAM (8.48 mmol), and without EtOH. The stirring time was 8 min, and the autoclave was heated 18 h at 180 °C. Measurements were performed on the ZEISS LIBRA 200FE microscope (ZEISS, Oberkochen, Germany) in transmission mode operated at 200 kV. For the determination of particle size, at least 200 particles were counted, and the statistical analysis of the images was performed with the software ImageJ (Version 1.43u).

**Formation of self-assembled TiO_2_ NP monolayers.** Self-assembled monolayer arrays of TiO_2_ NPs were formed following an approach within the literature [33]. TiO_2_ NPs prepared by solvothermal synthesis were dispersed in different non-polar solvents, or mixtures of the solvents, by ultrasonication. If the solution was turbid, despite continued ultrasonication, solutions of oleylamine or oleic acid in hexane were added until clear NP dispersions were obtained. A Teflon disk with a thickness of ca. 2 mm, an outer diameter of ca. 5 cm, and an inner circular hole with a diameter of ca. 2 cm was utilized for the self-assembly. The inner circular hole had to exhibit a sharp edge. The Teflon disk was placed on two 1 cm-high Al cubes standing in a Petri dish, and the whole setup was carefully leveled. Subsequently, tap water was filled into the Petri dish until the water surface contacted the underside of the disk. At this point, further tap water was slowly added with a Pasteur pipette until a concave upward curvature of the water surface was visible inside the inner circular hole of the Teflon disk. Drops of water were added until the water curvature changed to a slight convex upward curvature. The Petri dish was protected by a glass cylinder with a height of ca. 5 cm to minimize the influence of air currents on the surface. Approximately 0.4 mL of the NPs solution was gently dropped on the water surface, and the organic solvent was allowed to evaporate in the closed laboratory hood for 10 min. The air flow of the laboratory hood was measured with an anemometer. The colloid concentration, as well as the solvent or solvent mixtures, were investigated. The temperature and air velocity of the laboratory hood could not be controlled during the experiments. Best results were obtained with 0.56 mg/mL TiO_2_ NPs in 2.07 mL pentane, 0.85 mL DCM (100% purity, VWR Chemicals, Langenfeld, Germany), and 50 µL of 7.6 mmol oleylamine in hexane solution.

For TEM characterization of the self-assembled TiO_2_ NPs, the film on the water surface was lightly touched with the carbon-coated side of a carbon-coated cooper grid (S160, Plano, Wetzlar, Germany). Residual water was carefully removed with a tissue.

RS devices were prepared by two different approaches. For the one-step method, the TiO_2_ NP films floating on the water surface were gently touched with the Pt/Ir surface, and afterwards allowed to dry. For the other approach, a microcontact printing two-step method was applied from the literature. For the preparation of the PDMS stamp, glass microscope slides were placed in a plastic weighing dish and covered with Canada Balsam (Sigma Life Science, Taufkirchen, Germany) and nail polish 3 in 1 XXXL shine (Essence Multi Dimensions, Sulzbach, Germany). Subsequently, a silicon wafer with a native SiO_2_ oxide layer was glued to the glass substrate with the non-polished side, and dried for 30 min at 70 °C. Silicon oligomer and the catalyst of the SYLGARD^®^ 184 Silicone Elastomer Kit (Dow Corning, Wiesbaden, Germany) were mixed in a 10:1 weight ratio and filled into the weighing dish, covering the polished SiO_2_ surface, under stirring for ca. 10–15 min. After aging for 20 min, the polymerization process was continued at 70 °C for 3 h. Before usage, the PDMS stamps were peeled of the SiO_2_ wafer, and cut into the corresponding shape for the Pt/Ir electrodes. Directly before usage, the stamps were immersed into hexane, and subsequently in EtOH, for 5 min each, and dried with a stream of N_2_. A PDMS stamp was used to transfer the NP films from the water surface onto the Pt/Ir electrodes. The stamp was pressed lightly onto the films on the water surface, residual water was wiped off with a tissue, and finally, the stamp was pressed onto the Pt/Ir surface to transfer the TiO_2_ NP films. The transferred films on the Pt/Ir surface were characterized by AFM. Prior to the electrical characterization in SEM, the (Pt/Ir)/TiO_2_ NP film substrates were treated with O_2_ plasma to remove residual oleylamine or oleic acid ligands. The electrical characterization was performed by means of the nanorobotics setup in SEM, as well as by means of LC-AFM. The SEM chamber, and thus the measurement tips, as well as the TiO_2_ NP film, were treated with Ar plasma prior to the experiments.

**Preparation of Pt/Ir bottom electrodes.** Silicon wafers were cleaned in an ultrasonic bath with ultrapure water first, followed by EtOH, and then dried with N_2_. A Ti adhesion layer with a thickness of 10 nm, and a Pt/Ir (80:20) alloy layer with a thickness of approximately 160 nm, were deposited on the wafers by direct current (DC) sputtering (0.01 mbar Ar/100 W).

**Preparation of Pt/Ir coated tips.** AFM tips with a spring constant of approximately 40 N m^−1^ and with a special geometry were purchased from ATEC-NC, Nanosensors, Wetzlar, Germany. The front part of the cantilever is visible from the top, and thus can be monitored in the SEM. The tips were isotropically coated with Pt/Ir by radio frequency (RF) sputtering (0.017 mbar Ar/40 W). Metal-coated tips were freshly prepared before the measurements, and measured in the SEM to exclude contamination or damage of the tips. The obtained coated probes had a radius of curvature of approximately 100 nm.

**Electrical characterization with nanorobotics setup in SEM.** The electrical characterization was performed in situ in a field-emission scanning electron microscope ZEISS Supra 35-VP (ZEISS, Oberkochen, Germany) using a nanorobotics setup (Klocke Nanotechnik GmbH, Aachen, Germany) and a semiconductor analyzer (Agilent 4156C, bsw TestSystem & Consulting AG, Ismaning, Germany). Detailed information about the setup is given elsewhere [40]. Prior to the measurement, the electric conductivity of the tips was determined by contacting two tips with each other and measuring voltage sweeps from −10 mV → 10 mV → −10 mV. Experiments were only continued if a linear *I–V* behavior was observed, and a resistance below 1000 Ω was measured. Typically, the probe/probe resistance was ca. 400–600 Ω. Additionally, before addressing a TiO_2_ NP film spot, the probe was brought into contact with the Pt/Ir bottom electrode. Again, measurements were only continued if a linear *I–V* behavior was observed, and a resistance below 1000 Ω was measured. This control was repeated during the measurements. The voltage was applied to the Pt/Ir tip electrode, while the Pt/Ir film was grounded, and voltage sweeps from 0 V → *X* V → 0 V → −*X* V → 0 V, 0 V → −*X* V → 0 V → *X* V → 0 V were applied under high vacuum conditions (10^−6^ mbar). *I–V* curves were recorded with a current compliance (CC) to protect the metal coating of the tip electrode. Voltages and CC were varied during the experiments.

**Electrical characterization with local conductive atomic force microscopy.** LC-AFM measurements were performed at ambient pressure with a Cypher AFM from Asylum Research, Wiesbaden, Germany. Conductive diamond tips AppNano Doped Diamond with a radius of curvature of 100–300 nm were utilized.

## Supplementary Materials

The following are available online at www.mdpi.com/2079-4991/7/11/370/s1, Figure S1: Histograms of the synthesized TiO_2_ NPs’ longitudinal and transversal (a,b), Figure S2: Exemplary TEM images of the synthesized TiO_2_ NPs (a,b) and corresponding histograms of NPs’ longitudinal (7.9 ± 2.2) nm and transversal (4.8 ± 0.8) nm (c,d), resulting in a mean particle diameter of (6.3 ± 1.9) nm. Powder XRD patterns of the NPs (black) and literature anatase data (red) (e) [38], Figure S3: Exemplary TEM images of the self-assembled TiO_2_ NP film (a,b). Tapping mode AFM images of the TiO_2_ NP film transferred to a Pt/Ir surface by the one-step method. (c,d). Corresponding height profile (e) taken along the white line in (d) showing the height differences of approximately 8 nm, Figure S4: SEM image without enhanced contrast of a TiO_2_ NP film on the Pt/Ir surface, and on the left-hand side, the Pt/Ir coated tip electrode is visible, Figure S5: Three consecutive switching cycles recorded on a TiO_2_ NP monolayer (a), and subsequent permanent LRS (b), Figure S6: SEM image of a TiO_2_ NP layer after a SET process. The tip was lifted off the TiO_2_ NP layer, revealing a morphologicaly change of the layer.
