# Supplementary material for: Resistive Switching of Sub-10 nm TiO2 Nanoparticle Self-Assembled Monolayers
Source: Nanomaterials (Basel). 2017 Nov 4;7(11):370. doi: 10.3390/nano7110370 (PMC5707587; doi:10.3390/nano7110370)
Supplement: Supplementary file 1 [file nanomaterials-07-00370-s001.pdf]

Resistive Switching of sub-10 nm TiO_2 Nanoparticle Self-Assembled Monolayers

Dirk Oliver Schmidt [1,2], Nicolas Raab [3,4], Michael Noyong [1,2], Venugopal Santhanam [5], Regina Dittmann [3,4] and Ulrich Simon [1,2,*]

Supporting Information

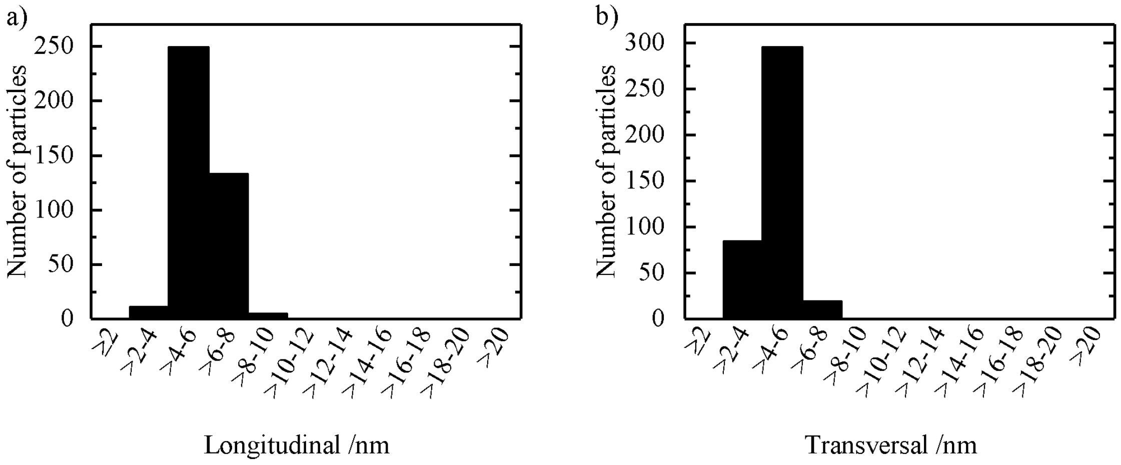

Figure S1. Histograms of the synthesized TiO_2 NPs′ longitudinal and transversal (a, b).

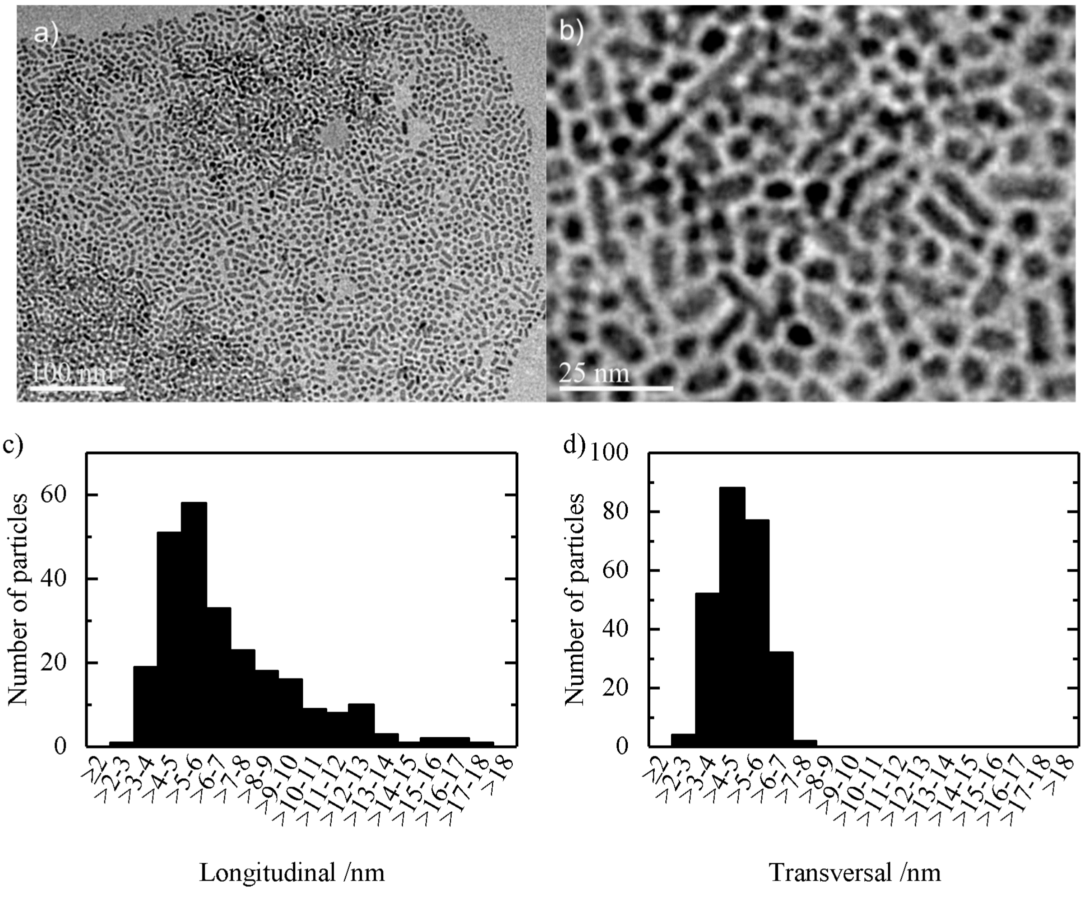

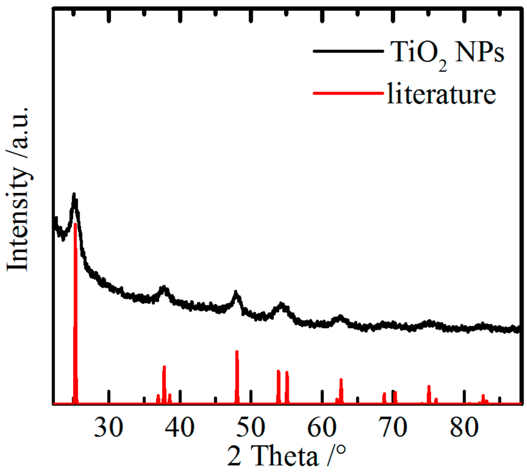

Figure S2. Exemplary TEM images of the synthesized TiO_2 NPs (a, b) and corresponding histograms of NPs' longitudinal (7.9 ± 2.2) nm and transversal (4.8 ± 0.8) nm (c, d), resulting in a mean particle diameter of (6.3 ± 1.9) nm. Powder XRD patterns of the NPs (black) and literature anatase data (red) (e) [38].

A self-assembled TiO_2 NP film was formed consisting of particles with a mean longitudinal of (9.5 ± 1.1) nm and mean transversal of (7.5 ± 0.9) nm and a spherical NP to non-spherical shaped NP ratio of 13:1. For TEM investigations, a small part of the self-assembled NP film was transferred onto a carbon coated TEM grid. The images revealed that mono-, bi- and multilayers were present as well as voids without any NPs (Figure S 3). For the one-step transfer method, the Pt/Ir substrate was gently brought into contact with the self-assembled film floating on the water surface (see Figure 2 d I and II in the main text) as compared the carbon grid. After evaporation of water residues, the TiO_2 NP film on the Pt/Ir surface was analyzed *via* AFM. As in the TEM images void spaces without any NP films were discernible in AFM images and multiple layers with various height differences were visible on the Pt/Ir surface (Figure S 4 c, d). A height profile was recorded along the white line in Figure S 4 d revealing a stepwise variation in height of approximately 8 nm. This height difference corresponds to the mean dimensions of the TiO_2 NPs of the mean longitudinal (9.5 ± 1.1) nm and mean transversal (7.5 ± 0.9) nm. Therefore, we assume that the first height step corresponds to a TiO_2 NP monolayer, the second step to a TiO_2 bilayer and so on.

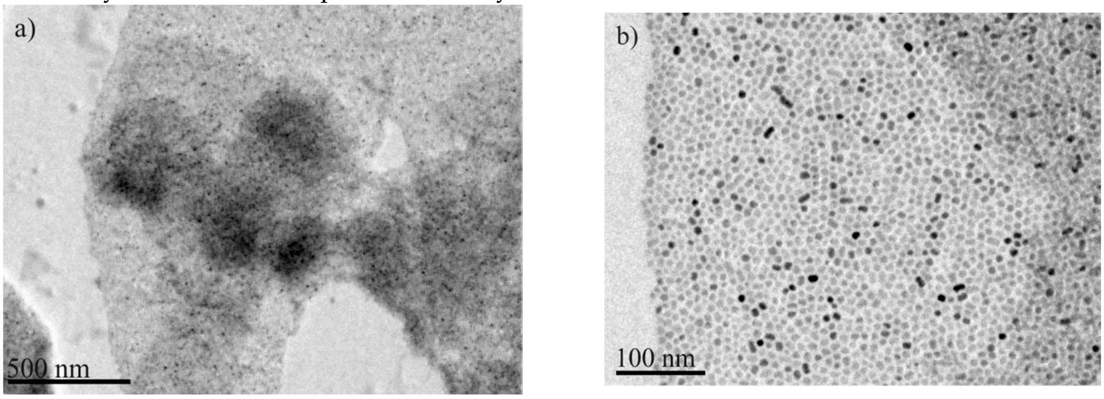

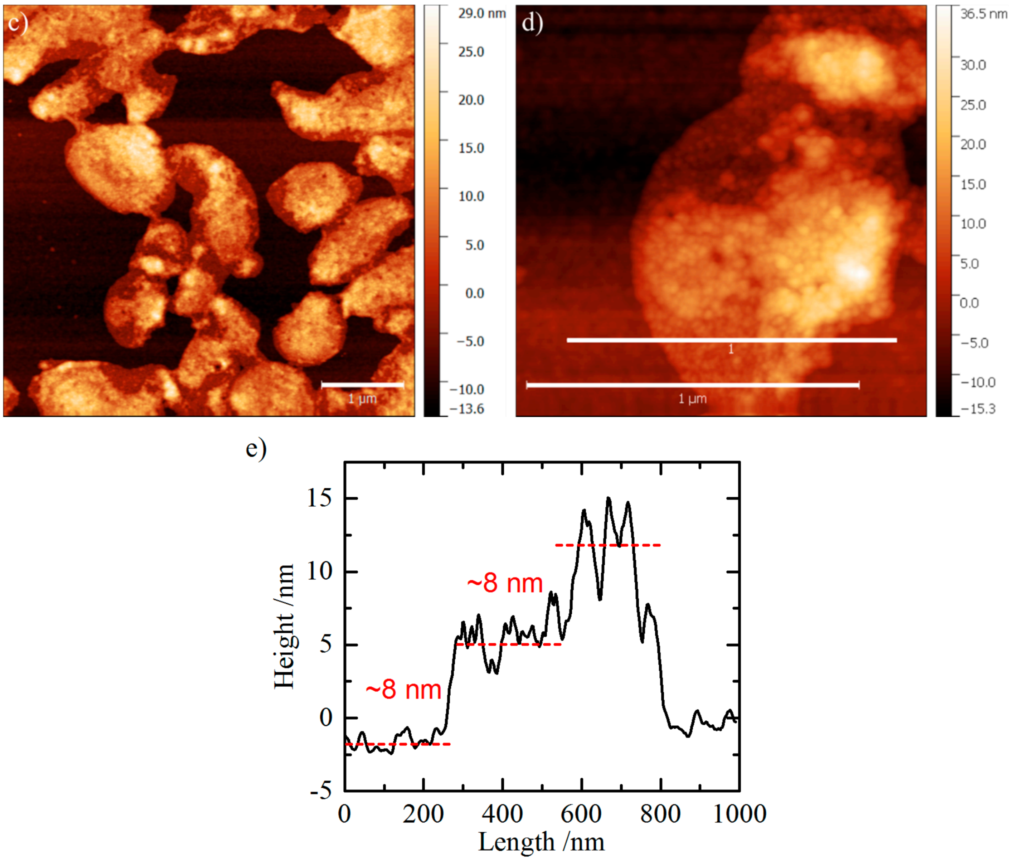

Figure S3. Exemplary TEM images of the self-assembled TiO_2 NP film (a, b). Tapping mode AFM images of the TiO_2 NP film transferred to a Pt/Ir surface by the one-step method. (c, d). Corresponding height profile (e) taken along the white line in (d) showing height differences of approximately 8 nm.

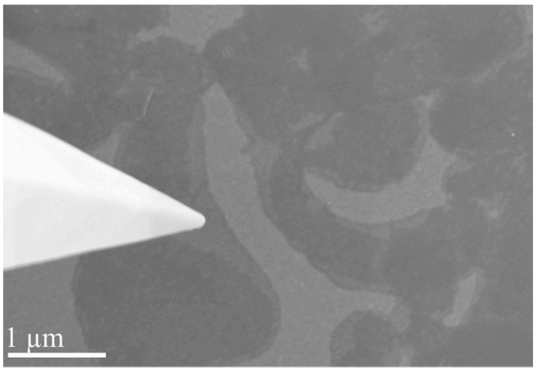

Figure S4. SEM image without enhanced contrast of a TiO_2 NP film on the Pt/Ir surface and on the left-hand side the Pt/Ir coated tip electrode is visible.

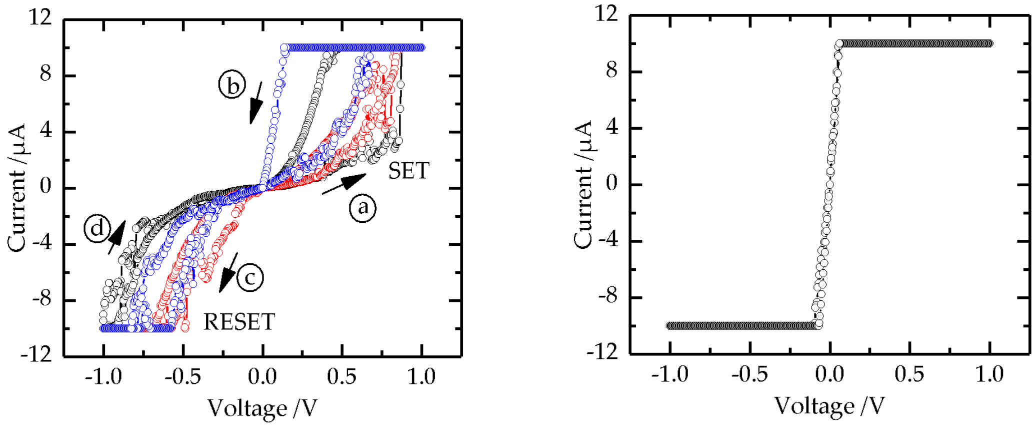

Figure S5. Three consecutive switching cycles recorded on a TiO_2 NP monolayer (a) and subsequent permanent LRS (b).

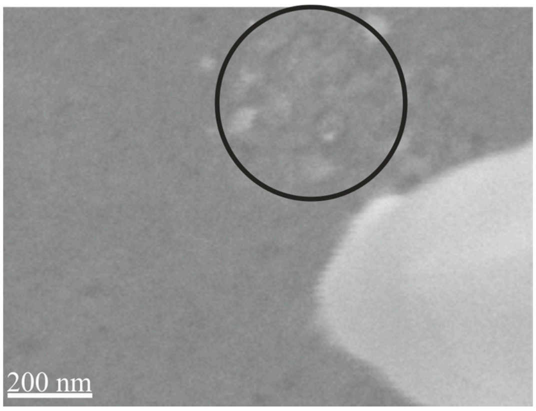

Figure S6. SEM image of a TiO_2 NP layer after a SET process. The tip was lifted off the TiO_2 NP layer, revealing a morphology change of the layer.
